# Association between dyslipidemia and cervical intraepithelial neoplasia: A case-control study in south-western Uganda

**DOI:** 10.4102/ajlm.v13i1.2374

**Published:** 2024-07-16

**Authors:** Frank Ssedyabane, Thomas C. Randall, Joseph Ngonzi, Rogers Kajabwangu, Alexcer Namuli, Joy Muhumuza, Josephine N. Najjuma, Deusdedit Tusubira

**Affiliations:** 1 Department of Medical Laboratory Science, Faculty of Medicine, Mbarara University of Science and Technology, Mbarara, Uganda; 2 Department of Global Health and Social Medicine, Massachusetts General Hospital, Harvard Medical School, Boston, Massachusetts, United States; 3 Department of Obstetrics and Gynaecology, Faculty of Medicine, Mbarara University of Science and Technology, Mbarara, Uganda; 4 Department of Obstetrics and Gynaecology, Mbarara Regional Referral Hospital, Mbarara, Uganda; 5 Department of Nursing, Faculty of Medicine, Mbarara University of Science and Technology, Mbarara, Uganda; 6 Department of Biochemistry, Faculty of Medicine, Mbarara University of Science and Technology, Mbarara, Uganda

**Keywords:** cervical intraepithelial neoplasia, dyslipidaemia, total cholesterol, low-density lipoprotein, high-density lipoprotein, triglycerides, Uganda, cervical cancer

## Abstract

**Background:**

Altered lipid levels may be associated with the development of a number of malignancies, including cancer of the cervix. However, there is limited understanding of this relationship in the rural Ugandan context.

**Objective:**

We investigated the connection between dyslipidaemias and cervical intraepithelial neoplasia (CIN) among women attending the cervical cancer clinic at Mbarara Regional Referral Hospital in south-western Uganda.

**Methods:**

This unmatched case-control study was conducted between December 2022 and February 2023 and included women with CIN (cases) and women without intraepithelial lesions (controls) in a 1:1 ratio. Participants were selected based on cytology and/or histology results, and after obtaining written informed consent. Demographic data were collected, and venous blood was drawn for lipid profile analysis. Dyslipidaemia was defined as: total cholesterol > 200 mg/dL, low-density lipoprotein > 160 mg/dL, triglycerides > 150 mg/dL, or high-density lipoprotein < 40 mg/dL. At diagnosis, cases were categorised as either CIN1 (low grade) or CIN2+ (high grade).

**Results:**

Among the 93 cases, 81 had CIN1, while 12 had CIN2+. Controls had a 13.9% (13/93) prevalence of high triglycerides and cases had a prevalence of 3.2% (3/93; *p* = 0.016). Reduced high-density lipoprotein was the most prevalent dyslipidaemia among cases (40.9%; 38/93). Statistically significant associations were found between high serum triglycerides and CIN (odds ratio: 1.395, 95% confidence interval: 0.084–1.851, *p* = 0.007).

**Conclusion:**

A notable association was observed between triglyceride dyslipidemia and CIN. Further studies into biochemical processes and interactions between lipids and cervical carcinogenesis are recommended through prospective cohort studies.

**What this study adds:**

This research provides additional information on the potential role of lipids in cervical carcinogenesis among women in rural Uganda. It also presents the possible prevalence of multimorbidity involving cervical cancer and cardiovascular diseases, particularly in low-resource settings lacking preventive measures against the increasing prevalence of dyslipidaemia.

## Introduction

Globally, there are 604 127 new cases of cervical cancer, ranking it as the fourth most common cancer, with 341 831 cervical cancer-related deaths worldwide in the year 2020.^1^ There has been an increasing prevalence of cervical cancer in Africa, especially the sub-Saharan region.^2^ It is the second most common cancer (with an incidence of 40.1/100 000 and mortality of 28.6/100 000), and the primary cause of cancer-related deaths among women in the East African region.^1^ Developed countries, implementing screening programmes for women aged 25 years and above, have witnessed a significant reduction in cases by more than a third, a trend not mirrored in less-developed nations,^3^ probably as a result of inadequate interventions, including vaccination against human papillomavirus (HPV).

It is a scientific fact that HPV is a necessary, albeit not standalone, factor in cervical cancer development.^4,5^ As a result, there is increasing research in the area of immunotherapy for advanced cervical cancer, especially the assessment of vaccine-related therapies, T-cell therapy and immune-checkpoint inhibitors in patients diagnosed with advanced cervical cancer.^6^ Cervical cancer development occurs through a number of stages, starting with a persistent infection with any of the HPV genotypes that are high risk, then cervical intraepithelial neoplasia (CIN), finally leading to invasive cervical cancer.^7,8^ Noteworthy cofactors, including HIV infection, *Chlamydia trachomatis* infection, smoking, gravidity, oral contraceptive use, and other forms of immunosuppression, also contribute to development of cervical cancer.^8,9,10,11^

Our prior study revealed a potential link between CIN and obesity, a component of metabolic syndrome.^12^ Other components of metabolic syndrome include dyslipidaemia and insulin resistance, which have been shown to be associated with treatment outcomes of cervical lesions.^13^ Lipids play a very critical role in metabolism, potentially serving as cofactors in the development of CIN and invasive cervical cancer.^14,15,16^ Alterations in lipids has been linked to development of a wide range of disease conditions.^17,18,19,20,21^ Moreover, dysregulated lipids have been shown to occur in development of malignancies,^16,17,22^ with recent evidence connecting disordered lipid levels to cervical cancer and its progression.^21,23^ Our previous study in an urban setting in Uganda showed a significant association between dyslipidaemia and cancerous cervical lesions.^24^

However, information on any potential association between lipid levels and CIN remains limited, especially in rural regions of Uganda. This study was therefore conducted to investigate whether there is an association between serum lipid levels and CIN among women attending the cervical cancer prevention clinic at Mbarara Regional Referral Hospital in south-western Uganda.

## Methods

### Ethical considerations

Ethical approval for this study was granted by the Mbarara University of Science and Technology Research Ethics Committee (MUST-2022-396) and the Uganda National Council for Science and Technology (HS2395ES). Additionally, administrative clearance from the Hospital Director at Hospital Director at Mbarara Regional Referral Hospital was sought before commencing the study. We obtained written informed consent from each participant and/or their legal guardian prior to their involvement in the study. In the case of illiterate participants, written informed consent was acquired from their authorised representatives. To maintain confidentiality, study numbers were used instead of names on all data collection tools, and participant identifiable information was separated during data analysis. Interactions between participants and the research team occurred in a private and comfortable setting, free from disturbances and accessible to only one participant at a time. Women diagnosed with cervical intraepithelial lesions (cases) received the standard care package at the cervical cancer clinic. The nurse at the clinic provided participants with their visual inspection with acetic acid, Pap smear, histology, and lipid profile results. All methods employed in this study adhered to relevant guidelines and regulations.

### Study setting

This study was conducted at the cervical cancer clinic of Mbarara Regional Referral Hospital, between 01 December 2022 and 27 February 2023. Mbarara Regional Referral Hospital is a regional referral hospital for the entire south-western Uganda and it is located in Mbarara city, approximately 260 km from the capital city, Kampala. This clinic offers services to an average of 75 women per week. It receives women from over 13 districts of the entire south-western region of Uganda plus the neighbouring countries including Tanzania, Rwanda and Democratic Republic of Congo.

### Study design

We conducted this unmatched case-control study including women who visited the Mbarara Regional Referral Hospital cervical cancer clinic. Cases were defined as women with a positive visual inspection with acetic acid result who underwent Pap smears and histology, subsequently confirmed to have CIN in accordance with the clinic’s standard care. Controls consisted of women with a negative visual inspection with acetic acid result and those confirmed to be devoid of cervical lesions based on cytology and histology.

### Sample size determination

We calculated our sample size using the online OpenEpi, Version 3, open-source calculator-SSCC (OpenEpi – Sample Size for Unmatched Case-Control Studies). For this calculation, we considered a two-sided confidence level of 95, a study power of 80% and a case-to-control ratio of 1. We took into account a proportion of cases with dyslipidaemia of 40.99%,^25^ along with a proportion of controls with dyslipidaemia of 18.8%,^26^ together with a least extreme odds ratio of 3.0. This is based on previous studies which have demonstrated a high variability in prevalence of dyslipidaemia in Africa, ranging from 5.2% to 89%.^27^ These studies offer the most accurate depiction of the cervical cancer study population that we could find.^25^ This gave a total of 75 cases and 75 controls using the Fleiss module with continuity correction.^28^ After factoring in the possible 15% attrition rate, the resultant sample size came to at least 86 cases and 86 controls.

### Sampling procedure

We prospectively selected cases and controls by using purposive sampling to recruit cases after cytology or histology specimen collection and following a positive screening test. Then we recruited a control each time a case was identified, that is, the incidence density sampling method as described previously.^29^

### Eligibility criteria

We included women aged 21 years and above, who visited the study site during the time of the study and who consented to participate. We excluded any woman who was undergoing any form of treatment with any lipid-altering drugs such as antihypertensive and cholesterol-lowering drugs.

### Data collection

#### Demographic data

Demographic information was gathered by use of a pretested enrollment form administered by research assistants, specifically the midwives from the Mbarara Regional Referral Hospital cervical cancer clinic. Upon obtaining written informed consent, participants received assistance in completing the enrollment form to record their demographic details. The collected data encompassed information such as age, district and village of residence, history of blood pressure and diabetes, marital status, highest attained level of education, HIV status, smoking habits, oral contraceptive use, family history of cervical cancer, age at sexual debut, number of lifetime sexual partners, and the number of full-term births.

#### Blood collection and lipid measurements

Four millilitres (4 mL) of venous blood was drawn aseptically by venipuncture from the mid-cubital vein, into plain vacutainers following consent and completion of the enrollment form. Specimens were given identification numbers (codes) and allowed to clot at room temperature, after which they were transferred to the laboratory for centrifugation (3000 revolutions per minute for 5 min) with the aim of separating serum from blood cells. The serum was then transferred into cryovial tubes using a micropipette. Lipid concentrations were measured using a fully automated analyser, Cobas 6000 Clinical Chemistry Analyzer (Roche Diagnostics, Indianapolis, Indiana, United States) following standard operating procedures.

#### Interpretation of lipid profile

Serum lipid measurements were interpreted as follows using adult reference ranges according to the National Cholesterol Education Program.^30^ We defined dyslipidaemia as total cholesterol concentration above 200 mg/dL, low-density lipoprotein (LDL) concentration above 160 mg/dL, triglyceride concentration of more than 150 mg/dL, or high-density lipoprotein (HDL) concentration below 40 mg/dL.

#### Histology and cytology

Using a cytobrush, cytological specimens were taken from the ectocervical and endocervical regions, smears made on spotless, marked glass slides and quickly fixed with 95% alcohol. For staining and analysis, fixed Pap smears were brought to the Mbarara University of Science and Technology histopathology lab. Using the Papanicolaou stains, all of the Pap smears were stained following standard operating procedures. The Principal Investigator and a pathologist performed the examination, and the Bethesda system for reporting cervical cytology^31^ was followed throughout the reporting process. Cone/punch biopsy samples were taken for histological analysis. After being fixed in 10% formaldehyde for 48 h, the samples were processed in an open system tissue processor for 14 h, following a standard operating procedure. After sectioning, Haematoxylin and Eosin staining was applied to the tissues. Stained slides were examined by the Principal Investigator together with a pathologist and reported as either CIN1, CIN2 or CIN3. CIN1 is considered ‘low grade’ and represents abnormal changes observed in less than one-third of the thickness of the cervical epithelium. CIN2 represents abnormal changes observed in between one-third and two-thirds of the thickness of the cervical epithelium, while CIN3 represents abnormal changes affecting more than two-thirds of the cervical epithelium. CIN2 and CIN3 are considered ‘high grade’.

### Data management and analysis

After collection by research assistants and the Principal Investigator, data were entered into an Excel spreadsheet (Microsoft Office Professional Plus 2013, version 15.0.4675.1003, Microsoft Inc., Redmond, Washington, United States) and then imported into STATA 13 (StataCorp LLC, College Station, Lakeway, Texas, United States) software. We used descriptive statistics including frequencies, means ± standard deviations, median values and interquartile ranges to characterise the population. Differences between respective groups were tested using the standard *t*-tests, Chi-square tests and the Fisher’s exact tests. Associations between CIN grades and specific dyslipidaemias were derived using logistic regression analysis. Cases with CIN2 or CIN3 were combined into a single group called ‘CIN2+’, because of the small number of cases in each group. All statistical outcomes were reported with 95% confidence intervals and *p*-values of < 0.05 were considered significant. We performed both bivariate and multivariate logistic regression analysis. Factors which were biologically plausible in cervical cancer and those whose *p*-values were ≤ 0.2 at bivariate analysis were considered for multivariate logistic regression analysis. These factors included age, smoking, contraceptive use, level of education, history of high blood pressure and history of diabetes.

## Results

### Study participants’ characteristics

In this case-control study, we enrolled 93 cases and 93 controls (Table 1). There was a statistically significant variation in age between the two groups (*p* < 0.001); the mean age of participants control group was 38.87 years and in the case group was 34.57 years. Additionally, more than half of the participants fell within the age group of 30 years – 49 years and this observation was statistically significant (*p* = 0.044). There were notable statistically significant differences in the highest level of education attained among study groups, with 13 out of 93 (13.9%) controls and 3 out of 93 cases (3.2%) reporting never having studied. Contraceptive use was reported by 38 out of 93 (40.9%) controls and 56 out of 93 (60.2%) cases. Among the cases, 7 out of 93 reported being smokers, resulting in a smoking prevalence of 7.6% among cases.

**TABLE 1:** Demographic characteristics of study participants at Mbarara Regional Referral Hospital between December 2022 and February 2023.

Variable	Category	CIN (*N* = 93)			No CIN (*N* = 93)			*p*
		Mean ± s.d.	*n*	%	Mean ± s.d.	*n*	%	
Age (years)†		34.57 ± 7.8	-	-	38.87 ± 8.3	-	-	< 0.001**
Age group (years)‡		-	-	-	-	-	-	0.044**
	20–29	-	24	25.8	-	16	17.2	-
	30–49	-	68	73.1	-	70	75.3	-
	50–59	-	1	1.1	-	7	7.5	-
Region of residency‡		-	-	-	-	-	-	0.740
	Central Uganda	-	2	2.1	-	1	2.2	-
	South-western Uganda	-	49	52.7	-	46	48.9	-
	Mbarara city	-	42	45.2	-	46	48.9	-
History of high blood pressure§		-	-	-	-	-	-	0.600
	No	-	74	79.0	-	71	76.0	-
	Yes	-	20	21.0	-	23	24.0	-
History of diabetes§		-	-	-	-	-	-	0.670
	No	-	81	87.1	-	79	84.9	-
	Yes	-	12	12.9	-	14	15.1	-
Marital status§		-	-	-	-	-	-	0.430
	Never married	-	21	227.0	-	14	15.1	-
	Married	-	53	56.9	-	60	64.5	-
	Divorced	-	19	20.4	-	18	19.4	-
Highest level of education‡		-	-	-	-	-	-	0.011**
	Never studied	-	3	3.2	-	13	13.9	-
	Preprimary	-	3	3.2	-	4	4.3	-
	Primary	-	45	48.5	-	35	37.6	-
	Secondary	-	25	26.9	-	34	36.6	-
	Tertiary	-	9	9.6	-	5	5.4	-
	University	-	8	8.6	-	1	2.2	-
HIV status‡		-	-	-	-	-	-	0.770
	Negative	-	50	53.8	-	52	55.9	-
	Positive	-	43	46.2	-	40	43.0	-
	Unknown	-	0	0.0	-	1	1.1	-
HIV viral load (copies/mL)†		18.9 ± 85.1	-	-	40.8 ± 161.3	-	-	0.400
CD4 count cells/UL†		488.2 ± 366.4	-	-	555.6 ± 362.3	-	-	0.700
Smoking‡		-	-	-	-	-	-	0.035**
	No	-	85	91.4	-	92	98.9	-
	Yes	-	7	7.6	-	1	1.1	-
Contraceptive use§		-	-	-	-	-	-	0.010**
	No	-	37	39.8	-	55	59.1	-
	Yes	-	56	60.2	-	38	40.9	-
Age at sexual debut (years)†		19.3 ± 3.8	-	-	18.4 ± 3.1	-	-	0.065

CIN, cervical intraepithelial neoplasia; s.d., standard deviation.

** , statistically significant.

† , independent *t*-test;

‡ , Fisher’s exact test;

§ , Chi-square test.

### Distribution of dyslipidaemias across cervical intraepithelial neoplasia grades

Out of the 93 cases, 81 had CIN1 while 12 had CIN2+. The mean triglyceride levels among cases was 95.9 mg/dL and among controls was 123.7 mg/dL, demonstrating a statistically significant difference (*p* = 0.001). High triglycerides showed a prevalence of 13.9% (12/93) among the controls and a lower prevalence of 3.7% (3/81) among cases, though this difference in distribution was not statistically significant (*p* = 0.08). High LDL showed a prevalence of 12.9% (12/93) among controls and a prevalence of 3.7% (3/81) among cases, and this distribution was also not statistically significant (*p* = 0.08). The prevalence of low HDL was higher in CIN2+ cases (66.7%; 8/12) although not statistically significant when compared to controls (*p* = 0.086) (Table 2).

**TABLE 2:** Distribution of dyslipidaemias across cytology/histology results of study participants at Mbarara Regional Referral Hospital between December 2022 and February 2023.

Variable	Category	Cases						Controls (*N* = 93)			*p*
		CIN2+ (*N* = 12)			CIN1 (*N* = 81)						
		Mean ± s.d.	*n*	%	Mean ± s.d.	*n*	%	Mean ± s.d.	*n*	%	
Serum HDL (mg/dL)†		43.6 ± 8.1	-	-	41.7.3 ± 8.1	-	-	44.7 ± 8.9	-	-	0.350
Serum LDL (mg/dL)†		77.9 ± 59.4	-	-	78.6 ± 28.7	-	-	148.6 ± 64.4	-	-	0.320
Serum total cholesterol (mg/dL)†		146.5 ± 36.7	-	-	148.3 ± 35.8	-	-	152.5 ± 43.2	-	-	0.390
Serum triglycerides (mg/dL)†		96.1 ± 49.4	-	-	94.9 ± 51.7	-	-	123.7 ± 59.6	-	-	0.001**
High total cholesterol‡		-	-	-	-	-	-	-	-	-	0.780
	No	-	12	100.0	-	75	92.6	-	85	91.4	-
	Yes	-	0	0.0	-	6	7.4	-	8	8.6	-
High triglycerides‡		-	-	-	-	-	-	-	-	-	0.080
	No	-	12	100.0	-	78	96.3	-	81	86.1	-
	Yes	-	0	0.0	-	3	3.7	-	12	13.9	-
Low HDL‡		-	-	-	-	-	-	-	-	-	0.086
	No	-	4	33.3	-	51	62.9	-	60	64.5	-
	Yes	-	8	66.7	-	30	37.1	-	33	35.5	-
High LDL‡		-	-	-	-	-	-	-	-	-	0.080
	No	-	12	100.0	-	78	96.3	-	81	87.1	-
	Yes	-	0	0.0	-	3	3.7	-	12	12.9	-

HDL, high-density lipoprotein; LDL, low-density lipoprotein; CIN, cervical intraepithelial neoplasia; s.d., standard deviation.

** , statistically significant.

† , independent *t*-test;

‡ , Fisher’s exact test.

### Association between dyslipidaemias and cervical intraepithelial neoplasia

From univariate logistic regression analysis, both high serum triglycerides and high serum LDL were found to be associated with CIN (Table 3). However, in multivariate logistic regression analysis, with adjustments made for age, smoking, contraceptive use, level of education, history of high blood pressure and diabetes, only high serum triglycerides was significantly associated with CIN (odds ratio: 1.395, 95% confidence interval: 0.084–1.851, *p* = 0.007). On the other hand, high serum LDL showed borderline association with CIN (odds ratio: 0.251, 95% confidence interval: 0.061–1.047, *p* = 0.058).

**TABLE 3:** Logistic regression analysis showing association between dyslipidaemias and cervical intraepithelial neoplasia among study participants at Mbarara Regional Referral Hospital between December 2022 and February 2023.

Variable	Category	Bivariate analysis			Multivariate analysis		
		Odds ratio	95% CI	*p*	Odds ratio	95% CI	*p*
High total cholesterol	No	1.000	-	-	1.000	-	-
	Yes	0.644	0.219–1.887	0.418	0.969	0.265–3.541	0.076
High triglycerides	No	1.000	-	-	1.000	-	-
	Yes	0.205	0.057–0.747	0.007	1.395	0.084–1.851	0.007**
Low HDL	No	1.000	-	-	1.000	-	-
	Yes	1.311	0.727–2.364	0.368	1.386	0.699–2.746	0.564
High LDL	No	1.000	-	-	1.000	-	-
	Yes	0.205	0.057–0.747	0.007	0.251	0.061–1.047	0.058

Note: *p*-values were obtained after adjusting for age, smoking, contraceptive use, level of education, history of high blood pressure, and history of diabetes.

HDL, high-density lipoprotein; LDL, low-density lipoprotein; CI, confidence interval.

** , statistically significant.

## Discussion

We present a high prevalence of reduced HDL in CIN (40.9%), especially those with CIN2+ (66.7%). We also present the overall prevalence of increased serum triglycerides as 3.2% in CIN and 3.7% specifically for CIN1. High serum triglycerides was also significantly associated with CIN in our study population.

Dyslipidaemia entails a variety of lipid abnormalities and may involve a combination of: extremely high cholesterol level of above 200 mg/dL, LDL cholesterol greater than 160 mg/dL, hypertriglyceridaemia of more than 150 mg/dL or significantly reduced HDL cholesterol of ≤ 40 mg/dL.^32^ It is one of the five metabolic syndrome components^33^ and it increases one’s risk of developing cardiovascular and other non-communicable diseases, including cancer of the cervix.^34^ It is therefore important to detect metabolic syndrome components in CIN in order to prevent heart and other cardiovascular diseases.

From our study, we observed that reduced HDL cholesterol was the most prevalent dyslipidaemia, an observation that has been reported in many previous studies.^35,36^ A study among South Africans in the Vaal region, between 2008 and 2011, reported similar findings on reduced HDL and increased triglycerides.^37^ A systematic review in 2018 revealed a reduced HDL prevalence of 41.4% and a prevalence of increased triglycerides of 16.5% in the general African adult population which is consistent with our findings.^38^ This shows an increased risk of cardiovascular diseases among the study population.

We did not observe any significant association between reduced HDL cholesterol and CIN. This is contrary to previous findings. For instance, Frontela-Noda et al., through their study at Cuba’s National Institute of Oncology and Radiobiology, among adult women from 2018 to 2020, reported a significant association between High Grade Squamous Intraepithelial Lesion and low HDL cholesterol.^39^ However, from a survey conducted from 1999 to 2010, a reduced serum HDL concentration was reported to be positively correlated with cervical cancer among United States citizens.^40^ This also indicated an increased likelihood of development of cardiovascular diseases among the study population.

This study found a statistically significant association between increased serum triglycerides and CIN. This observation is consistent with previous reports on the link between triglycerides and CIN. Though we studied CIN, a precursor stage for cervical cancer, previous cohort studies among Icelanders and Austrians showed that increased serum triglyceride concentration is associated with an increased cervical cancer prevalence.^41,42^ It is important, however, to note that observations in CIN cannot just be extrapolated to cervical cancer, even if one leads to the other. Another case-control study among United States adult women revealed that serum triglyceride concentrations are correlated with increased risk of cancer of the cervix.^40^ Dyslipidaemia is said to go hand in hand with other components of metabolic syndrome, including obesity, whose contribution to cervical cancer risk is not only reflected in elevated oestrogen levels^43^ but also in increased risk of HPV infection.^44,45^ Metabolic syndrome components are said to act as cofactors in cervical carcinogenesis, which is based on the positive correlation of oestrogen and adipokine levels with cervical cancer.^46,47^ Increased serum triglycerides and low HDL are said to be risk factors for cervical cancer.^48^ They are known to stimulate proliferation of cancer cells and induce anti apoptotic capacity by activation of reactive oxygen species.^49^

It is worth noting that a good fraction of our participants in both case and control groups reported a history of diabetes and hypertension. Previous studies have demonstrated that hypertension results in hypoxia, which results in synthesis of hypoxia-induced factor-1, which in turn promotes angiogenesis.^50^ It is hypoxia-induced factor-1 that is said to support development of HPV-induced lesions.^51^ The fact that controls also had a history of hypertension and diabetes, therefore, emphasises the multifactorial requirement for development of cervical cancer other than HPV infection.

This case-control study was designed with adequate power to derive associations. The other strength of this study was that we used internationally acceptable cut-off values for categorising dyslipidaemias. Also, the presence of CIN was confirmed with either Pap smear or histology, the gold standard, following national guidelines. For controls, absence of lesions was ascertained by a negative visual inspection with acetic acid test result, the routine screening test as recommended by national guidelines.

### Implications for clinical practice

This study demonstrates the association between triglyceride dyslipidaemia and CIN. With increasing prevalence of non-communicable diseases, patients would benefit from combined screening for cervical cancer as well as dyslipidaemia, preferably on a single visit. Just as for cervical cancer screening, a test and treat approach would be more impactful if coupled with screening for dyslipidaemia.

### Limitations

A major limitation of this study was the fact that controls were not matched with cases. Some confounders may still have had an effect on statistics. This further presents a shortcoming for case-control studies, in addition to their inability to ascertain causation. However, to the best of our knowledge, we gathered information on all factors known to be associated with cervical lesions and performed logistic regression analysis which generated a logistic regression model with all the factors included. We also expect our study to have suffered from selection bias as well as recall bias during participant enrollment. During collection of data, participants were required to remember information on a number of variables including history of diabetes and hypertension. These could have led to false conclusions about the study population since a lot of data may have been left out or improperly reported during data collection. However, for most of the variables that required participants to recall, we did our best to compare the provided information with participants’ charts. We also observe that the sample size used for this study was smaller than what is observed in other studies, despite the adequate power. This therefore might have a negative impact on the generalisability of results to the general population.

### Conclusion

We observed a significant association between dyslipidaemia, especially triglyceride dyslipidaemia, and CIN. This underscores the importance of expanding screening efforts for dyslipidaemia to effectively manage potential complications and other non-communicable diseases associated with dyslipidaemia. Furthermore, we advocate for prospective cohort studies, with larger sample sizes, to deepen our understanding of the relationship between dyslipidaemia and CIN, as well as its broader implications for cervical cancer. Adequate management of dyslipidaemia could stop progression of CIN to cervical cancer. Molecular studies could be carried out to understand the biochemical processes and interactions behind lipids and cervical carcinogenesis.
